# An Unusual Case of Finger Fracture

**DOI:** 10.7759/cureus.19577

**Published:** 2021-11-14

**Authors:** Leon Alexander

**Affiliations:** 1 Plastic & Reconstructive Surgery, Sheikh Khalifa Medical City, Abu Dhabi, ARE

**Keywords:** hand enchondromas, pathological fracture, curettage, recurrence, delayed approach, bone grafting

## Abstract

Enchondromas are commonly encountered primary bone tumors in the hand. They can be managed with various treatment options ranging from simple observation to curettage and bone grafting. In this study, the case of a finger enchondroma is reported; it presented as a pathological fracture. It was managed successfully using the delayed approach - initial stabilization of the fracture followed by definitive tumor clearance surgery. Concerning the early versus delayed approach used for fractures associated with enchondromas, there is an ongoing debate in the literature on whether to leave the cavity empty or fill it with bone graft after curettage. This article discusses the relevant literature on these issues and their impact on the refracture and rates of recurrence. Nevertheless, thorough curettage followed by regular monitoring in the follow-up period is important to minimize the morbidity associated with the recurrence of enchondromas.

## Introduction

Enchondromas are the commonest benign bone tumors of the hand. They present as innocuous hand swelling with minimal symptoms and are diagnosed primarily by radiographs. They can also present, albeit rarely, as multiple enchondromatosis - Ollier disease and Maffucci syndrome [1]. This article highlights the fact that enchondromas can present deceptively as a finger fracture following trivial trauma. They can be well managed by treating the fracture initially followed by tumor therapy.

There is a controversy in the literature on how to treat enchondromas of the hand, which occurs as a pathological fracture. Some authors recommend curettage of tumor and concurrent fracture fixation (early approach). In contrast, others recommend initial management of the fracture followed by definitive tumor clearance with a thorough curettage with/without bone grafting after the fracture has healed (delayed approach).

This report describes an enchondroma finger that presented as a pathological trauma following a minor injury. Initially, the fracture was treated by splinting followed by hand therapy. The patient was followed up regularly with serial radiographs. Subsequently, after six months, she underwent a thorough curettage followed by reconstruction with a bone graft. The treatment outcome was good, and there was no recurrence. The pros and cons of both treatment strategies (early versus delayed approach) are discussed in this study. However, irrespective of either approach, the local recurrence rate after curettage is 4.5%-7% [2].

## Case presentation

A 38-year-old female presented to us complaining of pain and stiffness of her left ring finger for six weeks. She had a history of fracture of the affected finger following a minor injury initially treated with splinting for six weeks at another hospital. Subsequently, she was treated with hand therapy, and serial radiographs were done regularly.

**Figure 1:**
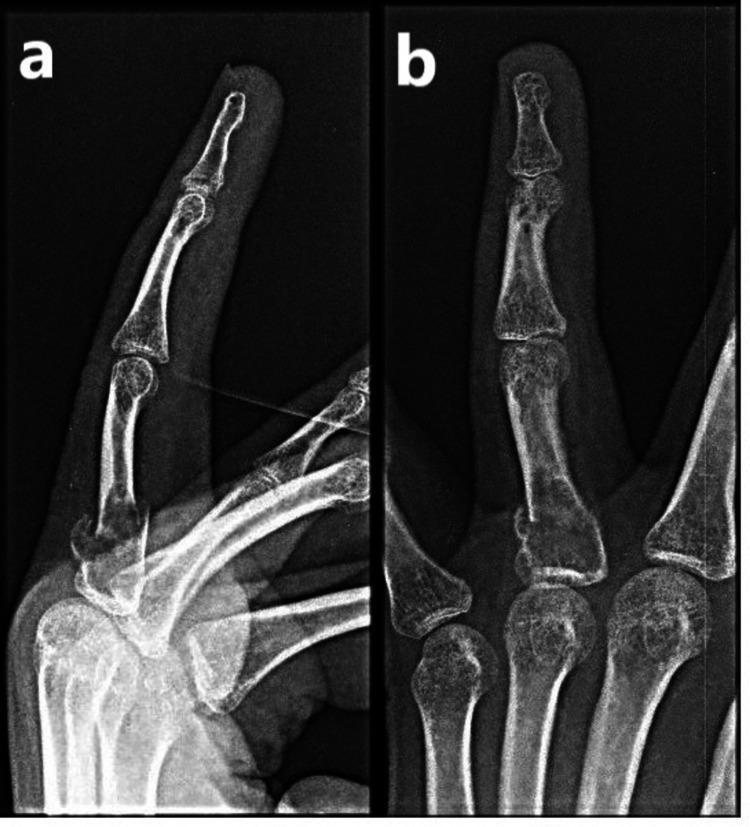
X-ray of the left ring finger. Lateral view (a) and oblique view (b) showing the fracture at the base of the proximal phalanx. Along with the fracture, there is an expansile lytic lesion seen in the metaphyseal region without cortical breach.

The lateral view (Figure 1a) and oblique view (Figure 1b) of the left ring finger show a fracture at the base of the proximal phalanx of the ring finger.

**Figure 2:**
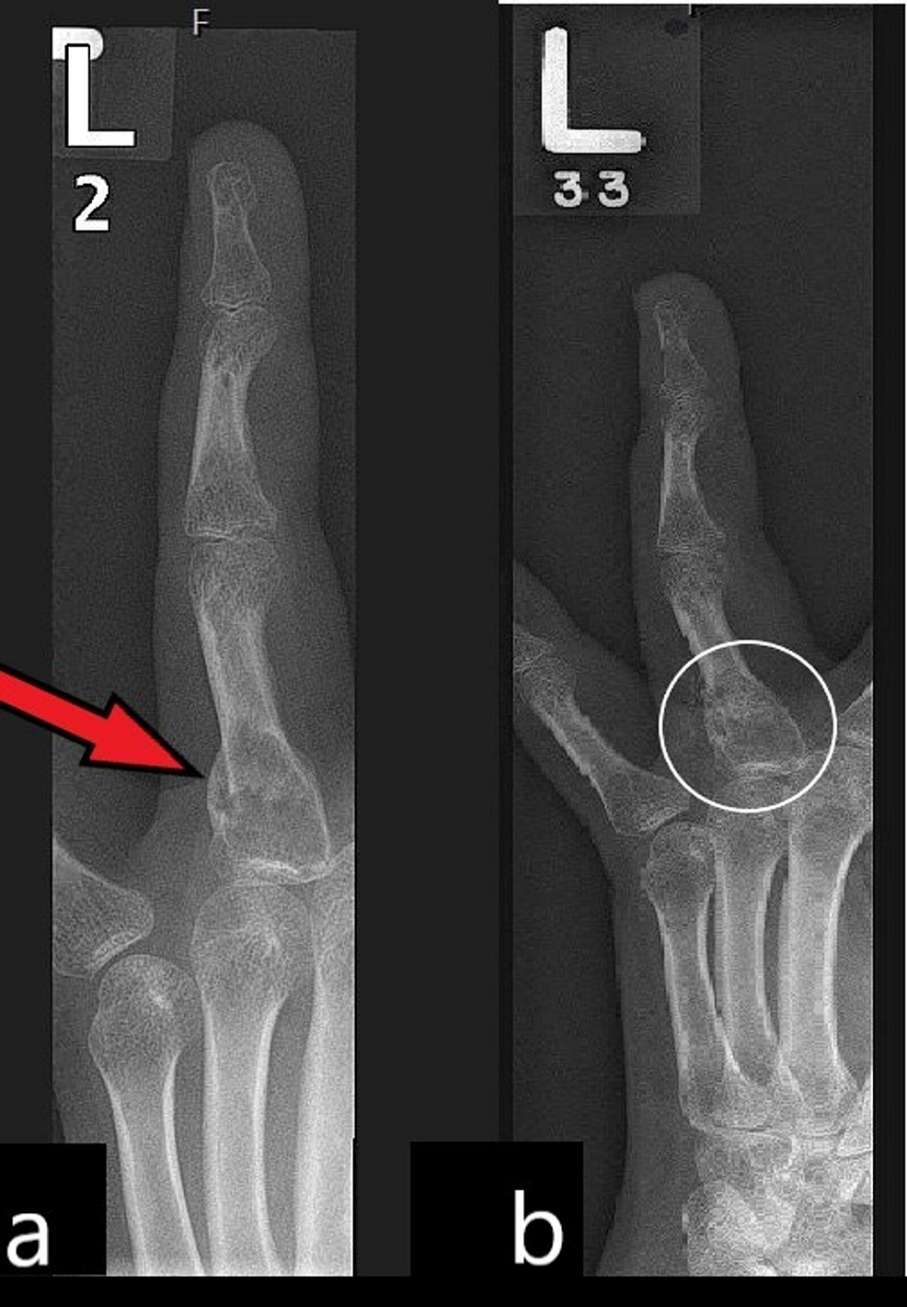
Follow-up radiographs of the left ring finger at two months showing (a) consolidation of the fracture at the base of the proximal phalanx (red arrow) and (b) almost complete bony union at six months (white arrow). The lytic lesion persists with sclerosis in the distal margin at the base of the proximal phalanx.

The fracture is seen through an abnormality in the metaphyseal region of the proximal phalanx with an associated nonaggressive expansile lytic lesion without cortical breach. There is no associated periosteal reaction, matrix calcification, or ossification. The subsequent follow-up images at two months (Figure 2a) show a near-complete consolidation of the fracture callus at the base of the proximal phalanx of the ring finger. Six months later (Figure 2b), there is a bony union at the fracture site. Still, the lytic lesion persists with sclerosis in the distal half of the lesion at six months.

**Figure 3:**
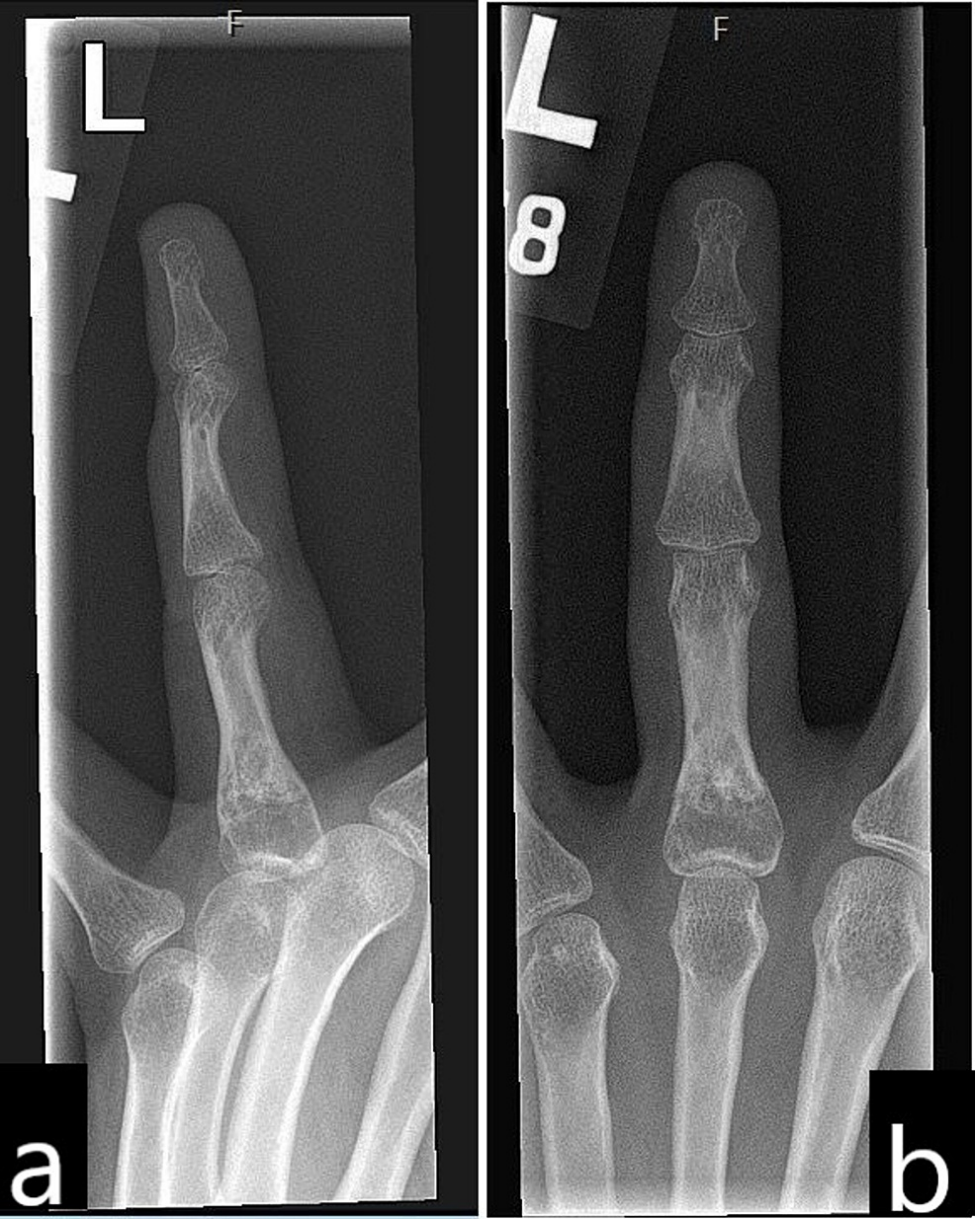
X-ray image at 15 months showing good incorporation of bone graft with no tumor recurrence.

After six months, she underwent a curettage of the enchondroma and reconstruction with a bone graft (freeze-dried allograft). Following surgery, the hand was splinted for six weeks, and hand therapy was started a few days after surgery. She regained almost complete range of motion at one year (total active motion (TAM): 240°), QuickDASH score of 15/100, and no tumor recurrence. The X-ray image at 15 months follow-up showed good bony consolidation and incorporation of bone graft with no recurrence of enchondroma (Figure 3).

## Discussion

Enchondromas are the commonest primary bone tumors arising in the hand and comprise 35%-65% of such tumors [1,2]. Commonly, enchondroma presents as a localized swelling with/without pain or as a pathological fracture following trivial trauma. The proximal phalanx is the most common bone involved in the hand, followed by the metacarpal and the middle phalanx. The carpal bones are rarely involved. Uncommon variants of enchondromas such as Ollier disease present with multiple enchondromas of the hands and feet. Maffucci syndrome is characterized by multiple enchondromas of the hands associated with hemangiomas. In both conditions, the risk of transformation to sarcoma (osteosarcoma and chondrosarcoma) is high [3]. The diagnosis is usually made with plain radiographs and confirmed by a tissue biopsy. X-ray imaging may be seen as a well-defined lytic lesion with lobulation and matrix calcification without cortical breach and soft tissue extension [4].

Small, asymptomatic tumors can be managed conservatively with serial radiographs and regular follow-up. However, large and symptomatic tumors with atypical radiographic features must be treated by biopsy and curettage. Planning for surgery entails placing the biopsy incision within the field of limb salvage incision or amputation flap markings. Once the frozen section biopsy confirms benign enchondroma, the next step is to proceed with the limb salvage incision for a thorough tumor curettage. The mid-lateral approach is preferred instead of the dorsal approach to minimize tumor contamination of the extensor mechanism, postoperative adhesions, and finger stiffness due to extensor tendons sticking to the bone [4].

When enchondromas present as a pathological fracture, the definitive treatment is deferred until the fracture has healed and entails thorough curettage and filling the cavity with allograft or autograft. To shed more light on the controversy of the early versus delayed approach in dealing with enchondromas presenting as pathological fractures, a table of comparative studies is presented (Table 1).

**Table 1:** Early versus delayed treatment of hand enchondromas.

Study (Year)	No. of Cases	Outcomes
Ablove et al. (2000) [5]	16	Better outcomes in the delayed group, higher complications in the early group – loss of motion and fracture displacement.
Sassoon et al. (2012) [6]	102	Similar outcomes in both groups in terms of the range of motion and time to full motion.
Whiteman et al. (1993) [7]	20	The delayed group had a faster return to motion.
Zhou et al. (2017) [8]	148	Early treatment had better outcomes.

In the case reported here, the fracture was managed conservatively with splinting followed by hand therapy. After six months, the patient underwent a curettage of the tumor and bone grafting. The functional outcome is better if treatment is delayed until after the fracture has healed as the presence of osteoid during fracture healing may lead to a diagnostic dilemma. Ablove et al., in their study on early versus delayed treatment of hand enchondromas, observed that the advantage of early treatment was a decrease in the disability period and a single-stage fracture fixation and tumor clearance surgery [5]. However, there was a higher complication rate in the immediate treatment group. They concluded that the immediate treatment option of hand enchondromas should be approached with caution for the reasons given above, and the functional outcome is better if definitive tumor curettage is deferred until the fracture has healed [9].

Other controversial aspects of managing hand enchondromas worth pondering include whether filling the cavity with a bone graft or spacer or leaving it empty after curettage has any long-term effect on the risk of refracture or local recurrence of the tumor. Bauer et al., in their study, used bone grafts, both autogenic iliac cancellous bone graft and cadaveric cortico-cancellous allograft, after curettage of hand enchondromas and reported similar good outcomes in both groups [9]. Yasuda et al., in their series, used bone cement as a graft to reconstruct the defect after tumor curettage, and there was partial resorption of bone cement in two out of the 10 patients [10]. Other studies have reported equally good outcomes when curettage is done without bone grafting [11,12]. Goto et al. suggested that when curettage without bone grafting is performed, it is important to replace the cortical window after curettage to hasten the recovery of the mechanical strength of the bone [12].

Unfortunately, the incidence of local recurrence after curettage reported in the literature is 4%-7%. In addition, malignant degeneration of enchondroma into chondrosarcoma has also been reported [2,5]. It is important to regularly follow-up patients after curettage to monitor for recurrence of enchondroma.

## Conclusions

Enchondromas are commonly encountered benign tumors of the hand. There are multiple treatment options for these tumors, but the basic principle is thorough curettage of the lesion followed by regular follow-up. Future research must be focused on the early versus delayed approach in managing enchondromas associated with fractures. Finally, comparing the various treatment options following curettage of these lesions (autograft versus allograft versus bone cement versus leaving the cavity empty) with multicenter studies will help formulate an optimal treatment protocol in managing these tumors.
